# Characterization of regulatory features of housekeeping and tissue-specific regulators within tissue regulatory networks

**DOI:** 10.1186/1752-0509-7-112

**Published:** 2013-10-31

**Authors:** Pengping Li, Xu Hua, Zhen Zhang, Jie Li, Jin Wang

**Affiliations:** 1 The State Key Laboratory of Pharmaceutical Biotechnology, Jiangsu Engineering Research Center for MicroRNA Biology and Biotechnology, School of Life Science, Nanjing University, Nanjing, China

**Keywords:** Regulatory networks, Tissue-specific genes, Housekeeping genes, Cluster coefficient

## Abstract

**Background:**

Transcription factors (TFs) and miRNAs are essential for the regulation of gene expression; however, the global view of human gene regulatory networks remains poorly understood. For example, how is the expression of so many genes regulated by limited cohorts of regulators and how are genes differentially expressed in different tissues despite the genetic code being the same in all tissues?

**Results:**

We analyzed the network properties of housekeeping and tissue-specific genes in gene regulatory networks from seven human tissues. Our results show that different classes of genes behave quite differently in these networks. Tissue-specific miRNAs show a higher average target number compared with non-tissue specific miRNAs, which indicates that tissue-specific miRNAs tend to regulate different sets of targets. Tissue-specific TFs exhibit higher in-degree, out-degree, cluster coefficient and betweenness values, indicating that they occupy central positions in the regulatory network and that they transfer genetic information from upstream genes to downstream genes more quickly than other TFs. Housekeeping TFs tend to have higher cluster coefficients compared with other genes that are neither housekeeping nor tissue specific, indicating that housekeeping TFs tend to regulate their targets synergistically. Several topological properties of disease-associated miRNAs and genes were found to be significantly different from those of non-disease-associated miRNAs and genes.

**Conclusions:**

Tissue-specific miRNAs, TFs and disease genes have particular topological properties within the transcriptional regulatory networks of the seven human tissues examined. The tendency of tissue-specific miRNAs to regulate different sets of genes shows that a particular tissue-specific miRNA and its target gene set may form a regulatory module to execute particular functions in the process of tissue differentiation. The regulatory patterns of tissue-specific TFs reflect their vital role in regulatory networks and their importance to biological functions in their respective tissues. The topological differences between disease and non-disease genes may aid the discovery of new disease genes or drug targets. Determining the network properties of these regulatory factors will help define the basic principles of human gene regulation and the molecular mechanisms of disease.

## Background

Gene regulation is underpinned by interactions among regulators, including transcription factors and miRNAs and their target genes. The elucidation of gene regulation is important for understanding the behaviour of biological systems and in recent years, network-based approaches have been widely used to uncover the mechanisms of gene regulation [1-4]. The elements involved and the structure of transcriptional regulatory networks reflect the behaviour of complex systems at the system-wide level [5]. It is also possible to infer how biological processes or functions change when the regulatory system confronts specific gene mutations, gene knockouts or pharmacological treatments [6].

Simple topological properties can directly reflect important cellular functions [7]. For example, different types of genes have different topological properties in human biological networks [8-11], and these properties can help to identify the functions of new genes. Genes with certain topological properties were found to have particular importance in the organization of human biological networks. For example, Han et al. [12] and Taylor et al. [13] found that removal of two classes of hubs could strongly affect the organization of protein interaction networks, while analysis of network dynamics for multiple processes by Luscombe et al. [14] revealed large topological changes in regulatory networks. Gerstein et al. [4] found that factors at different in-degree or out-degree levels in a regulatory network have different biological functions and Bhardwaj et al. [15-17] showed that factors with different hierarchies in model organism regulatory networks have different properties. Specifically, Lin et al. [18] revealed that housekeeping (HK) and tissue-specific (TS) proteins have their own structural organization in human protein interaction networks. In addition, tissue-specific genes were twice as likely as housekeeping genes to be drug targets, allowing the identification of tissue ‘signature networks’ that will facilitate the discovery of new therapeutic targets and biomarkers of tissue-targeted diseases [19]. miRNA studies also showed that functionally distinct classes of miRNAs have specific topologies in regulatory networks [2,20-22]. However, the mechanism by which HK and TS encoded regulators (TFs and miRNAs) are organized in gene human regulatory networks and the biological significance of the network properties await elucidation.

A central goal of our study was to classify how regulators are organized to realize gene expression patterns in different cell types and tissues. We have addressed this problem by constructing miRNA-TF regulatory networks for seven human tissues, including brain, heart, kidney, liver, ovary, spleen and testis. We have then analysed the regulatory features of these TFs and miRNAs through analysis of their topological properties in the regulatory network. We have also classified the regulatory structures of disease-related genes and miRNAs in the networks.

We divided TFs into three sets: HK TFs, TS TFs and trivial TFs and miRNAs into two sets: TS miRNAs and trivial miRNAs. We then compared the topological properties of HK/TS TFs with trivial TFs, TS miRNAs with trivial miRNAs, and HK TFs with TS TFs. In addition, we also determined the topological bias between disease TFs/miRNAs and non-disease TFs/miRNAs. Our study intends to provide a global view of the organization of housekeeping TFs, TS TFs and miRNAs in a gene transcriptional regulatory network and to uncover specific properties of disease genes and miRNAs in the network. This will further our understanding of miRNA and TF regulation and provide clues to identify human disease genes in the network.

## Results

We characterized the regulatory patterns of different types of genes by scaling their topological features in tissue regulatory networks. These networks comprise the regulatory interactions between TF, miRNA and non-TF genes (see Methods). The topological features of each gene (vertex) were quantified with four centrality measures, i.e. in-degree (the number of incoming edges to each vertex), out-degree (the number of outgoing edges from each vertex), betweenness (the percent of shortest paths that go through a vertex) and cluster coefficient (the percent of neighbours of a vertex that connect to each other) [23]. We detected the topological features of genes in seven tissue regulatory networks, and investigated the topological biases of the HK and tissue-specific TS genes (see Methods).

### Topological bias of TS/HK genes relative to trivial genes

Table 1 presents the global view of topological differences among HK and TS genes in the seven tissue regulatory networks relative to randomly selected trivial genes from the same network, i.e. non-HK and non-TS genes (see Methods).

**Table 1:** Significant topological bias of TS/HK genes relative to trivial genes

	**In-degree**	**Out-degree**	**CC**	**Btwn**
**Brain**				
**Heart**				
**Kidney**				
**Liver**				
**Ovary**				
**Spleen**				
**Testis**				

P < 0.10. cc: cluster coefficient; Btwn: betweenness, Red five-pointed star: TS miRNAs; Green five-pointed star: TS TF; Green diamond: HK TF.

Red five-pointed star indicates that the relative centralities of TS miRNAs are significantly higher than those of trivial miRNAs in the corresponding tissue. Green five-pointed star indicates that the relative centralities of TS TFs are significantly higher than those of trivial TFs in the corresponding tissue; Green diamond indicates that the relative centralities of HK TFs are significantly higher than those of trivial TFs in the corresponding tissue. P < 0.10.

### TS miRNAs

**Figure 1:**
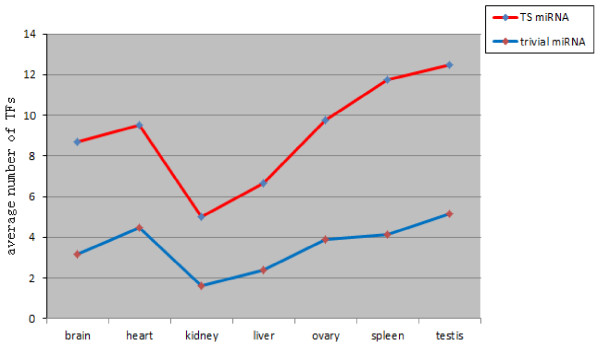
The average number of TFs that connect to TS miRNAs (red) and trivial miRNAs (blue) in seven tissues.

Compared with trivial miRNAs, TS miRNAs showed significantly higher cluster coefficients in most of the tissues (five out of seven). The cluster coefficient measures the connecting density of neighbours to a gene. The higher it is, the more densely the neighbours are connected. Thus, this suggests that TS miRNAs are likely to be involved in small, densely connected clusters. Furthermore, because neighbouring genes of the TS miRNAs have the tendency to connect to each other they are more likely to be TFs, because TFs are the only type of regulator that is capable of connecting with both miRNAs and its targets. Such deduction is supported by the detection of the number of TFs connecting TS and trivial miRNAs (see Figure 1). In all seven tissue regulatory networks, the numbers of TFs connecting to TS miRNAs was apparently larger than that connecting to trivial miRNAs. The local cluster behaviour of TS miRNAs and TFs also suggests strong co-regulation of gene expression by TS miRNAs and TFs.

**Figure 2:**
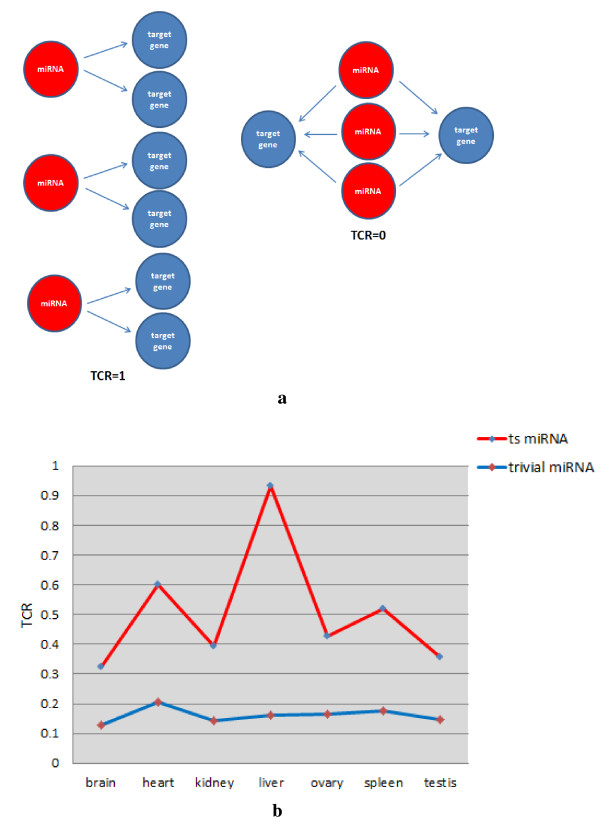
**TS miRNAs show higher TCR values compared with trivial miRNAs. (a)** The TCR for three miRNAs in the left network is 1, while the TCR for three miRNAs in the right network is 0. (**b**) The TCRs for TS miRNAs (red) and trivial miRNAs (blue) in seven tissues.

**Figure 3:**
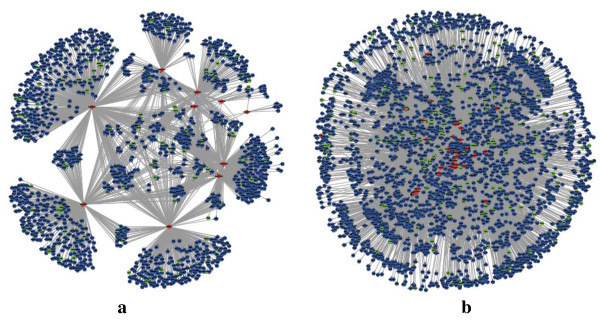
**Examples of sub-networks in the heart.** (**a**) Sub-network in which regulators are TS miRNAs. (**b**) Sub-network in which regulators are trivial miRNAs. miRNAs are shown as yellow circles, while TFs and non-TFs are indicated by green and blue circles, respectively. miRNAs are denoted by yellow circles.

Interestingly, although the average out-degree of TS miRNAs was comparable to that of trivial miRNAs (no significance in Table 1), the average global target number of TS miRNAs was higher than that of trivial miRNAs across all seven tissues. This led to the high target compact rate (TCR) of TS miRNAs relative to trivial miRNAs (Figure 2). The TCR is defined as:

(1) $$\mathrm{TCR}=\frac{{<N}_{\mathrm{targ}}{>/<K}_{\mathrm{out}}>-{1/N}_{\mathrm{mir}}}{1-{1/N}_{\mathrm{mir}}}$$

where < *N*_*targ*_ > and < *K*_*out*_ > are the average target number and average degree respectively, and *N*_*mir*_ is the number of miRNAs. TCR equals 0 when all the out-degrees converge to the same target, and equals 1 when every out-degree directs to different targets (Figure 2a). Such behaviour (Figure 2b) suggests that TS miRNAs tend to regulate different sets of targets, whereas the trivial miRNAs tend to co-regulate the same set of targets. Such distinct regulatory features of TS and trivial miRNAs were also observed in the shape of the related graphs. Taking the heart as an example, the sub-network composed of TS miRNAs and their targets was assembled by several small fans, where the root is the TS miRNA and the leaves are the specific targets of the TS miRNA (Figure 3). However, the sub-network composed of trivial miRNAs and their targets behaved chaotically, with trivial miRNAs and their targets mixed together.

### TS TFs

**Figure 4:**
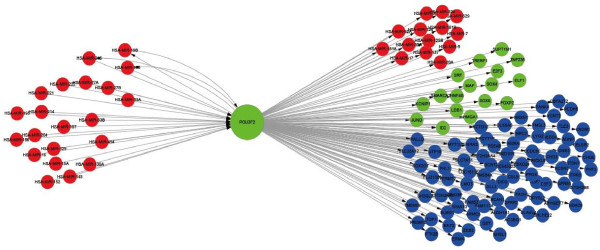
**A subnet formed by POU3F2 and its targets and miRNAs that regulate POU3F2.** Target TFs of POU3F2 are denoted by green circles and target non-TFs by blue circles; red circles are miRNAs. Arrows represent transcriptional regulation from regulators to target genes.

In four out of the seven tissues (brain, kidney, liver and ovary), all four topological parameters (i.e. in-/out- degree, cluster coefficient and betweenness) of TS TFs were significantly higher compared with those of the trivial TFs (Table 1). The high in-/out-degree indicates that TS TFs tend to regulate a large number of targets, and that TS TFs are regulated by a large number of regulators, while the high betweenness values show that TS TFs locate to bridge positions through which many regulatory pathways pass. These two features suggest that TS TFs act as information-transition hubs in either the local or global topology of tissue regulatory networks. The TS TFs receive a large amount of information from the upstream regulators (or pathways), and then re-distribute the information to a large number of downstream targets (or pathways). A deficiency of TS TFs would severely disrupt the transition of regulatory information, and lead to dysfunction of associated biological processes. For example, POU3F2, a brain TS TF, has high in-degree, out-degree, betweenness and cluster coefficient values. It regulates 115 genes (among which 17 are TFs and 17 are miRNAs). At the same time it is heavily regulated by 21 miRNAs. Among these 21 miRNAs, three mutually regulate POU3F2, and also regulate some of the targets of POU3F2 (Figure 4). GeneCards [24] documents the involvement of POU3F2 in the neurogenesis pathway and in SIDS (Sudden Infant Death Syndrome) susceptibility pathways. In neurogenesis, POU3F2 is activated by association with other POU gene products and alters the expression of banks of downstream genes that participate in specification of neuronal cell subsets in the brain [25]. POU3F2 also regulates the transcription of a diverse set of genes in the central nervous system and regulates stem cell state [26]. These observations indicate that POU3F2 occupies a vital position in the transmission of regulatory signals in a brain regulatory network and plays essential roles in neuronal differentiation by promoting morphogenesis of neuronal cells.

A high cluster coefficient suggests that TS TFs are preferred to regulate/be regulated by regulators in the same way as miRNAs, as described above. The difference is that the regulator that is able to interact with a miRNA can only be a TF. Here, the regulator that interacts with a TS TF could be a miRNA as well as a TF. Both TFs and miRNAs are significantly enriched in the neighbours of TS TFs (P-value < 0.05, Fisher exact test, see Table S1 in Additional file 1) across all the tissues. Moreover, the number of neighbours that are miRNAs is significantly higher compared with that of TFs (Fisher exact test, P-value < 0.05, see Table S2 in Additional file 1), suggesting a particularly strong co-regulation of TS TFs and miRNAs on gene expression.

Significantly high out-degree values for TS TFs were found in the heart and spleen, whereas significantly high betweenness values for TS TFs were observed in the brain and spleen. This may be related to the specific topological features of TS TFs in distinct tissues.

### HK TFs

In the majority of tissues (six out of seven), the HK TF cluster coefficients were significantly higher than those of trivial TFs. (Table 1), suggesting that HK is associated with local, densely connected clusters and that TFs tend to regulate/be regulated by other TFs and miRNAs, as described above for TS TFs (Fisher exact test, P-value < 0.05, see Table S3 and Table S4 in Additional file 1). Specifically, HK TFs show significantly high out-degree values in two tissues, kidney and spleen.

### Topological bias for TS genes relative to HK genes

We also compared the centrality topology of TS genes and HK genes (Table 2). Because there are no HK miRNAs (no miRNA has been found to be widely expressed throughout different tissues, see Methods), only the topological bias of TS TFs relative to HK TFs was detected.

**Table 2:** Significant topological bias of TS TFs relative to HK TFs

	**In-degree**	**Out-degree**	**CC**	**Btwn**
**Brain**				
**Heart**				
**Kidney**				
**Liver**				
**Ovary**				
**Spleen**				
**Testis**				

P < 0.10. cc: cluster coefficient; Btwn: betweenness, Green five-pointed star: TS TF.

Green five-pointed star indicates that the relative centralities of TS TFs are significantly higher than those of HK TFs in the corresponding tissue. P < 0.10.

In kidney and ovary, the out-degree, cluster coefficient and betweenness values of TS TFs were all significantly higher compared with those of HK TFs (Table 2). The out-degree and betweenness values of TS TFs relative to those of HK TFs could be inferred, because TS TFs showed significantly high out-degree/betweenness values relative to those of trivial TFs, whereas no significant difference was found between the out-degree/betweenness values of HK TFs and trivial TFs for these two tissues (Table 1). However, TS TFs and HK TFs both showed significantly higher cluster coefficients relative to trivial TFs (Table 1). Thus, a distinct order was determined with TS TFs having the highest cluster coefficients, followed by HK TFs and trivial TFs having the lowest.

There were also some features that were only present in specific tissues (Table 2). For example, in brain the TS TF in-degree value was higher than that for HK TFs. In addition, high cluster coefficient/betweenness values for TS TFs were observed in the heart and brain.

### Topological bias of disease genes relative to non-disease genes

Numerous disease genes are expressed in all of the seven tissues. In each tissue, the number of disease TFs was comparable to that of non-disease TFs (see Additional file 1: Table S2). No tendency of disease TFs to be either TS TFs or HK TFs was found (Fisher test, P-value >0.5). However, the cluster coefficients of disease TFs were significantly higher compared with those of non-disease TFs (Table 3), suggesting that disease TFs tend to be involved in small clusters that mainly consist of regulators. In addition to the TS and HK TFs mentioned above, both TFs and miRNAs were significantly enriched in disease TF neighbours (P-value < 0.05, Fisher exact test, see Table S5 in Additional file 1) across all the tissues. Moreover, the number of neighbours that were miRNAs was significantly higher than the number that was TFs (Fisher exact test, P-value < 0.05, see Table S6 in Additional file 1), suggesting particularly strong co-regulation by disease TFs and miRNAs on gene expression. In addition, significantly high out-degree values of disease TFs were specifically found in heart and liver.

**Table 3:** Significant topological bias of disease genes relative to non-disease genes

	**In-degree**	**Out-degree**	**CC**	**Btwn**
**Brain**				
**Heart**				
**Kidney**				
**Liver**				
**Ovary**				
**Spleen**				
**Testis**				

P < 0.10. cc: cluster coefficient; Btwn: betweenness, Red circle: disease miRNA; Green circle: disease TF.

Red circle indicates that the relative centralities of disease miRNAs are significantly higher than those of non-disease miRNAs in the corresponding tissue. Green circle indicates that the relative centralities of disease TFs are significantly higher than those of non-disease TFs in the corresponding tissue. P < 0.10.

The disease miRNAs constituted more than 80% of miRNAs in every tissue (see Table S7 in Additional file 2). Both TS and trivial miRNAs could be disease associated. No significant tendency of disease miRNAs to be TS miRNAs was identified in any of the seven tissues (Fisher test, P-value >0.5). Nevertheless, there were distinct differences in topology between disease miRNAs and non-disease miRNAs. In all seven tissues, the out-degree values of disease miRNAs were significantly higher than that of non-disease miRNAs (Table 3), suggesting that the disease miRNAs tend to co-ordinately regulate a large number of targets. Moreover, the disease miRNAs also showed tissue-specific features in certain tissues. For example, the in-degree values of disease miRNAs were relatively higher in brain, kidney, liver and testis, whereas the betweenness values of disease miRNAs were high in kidney, liver, ovary and testis.

## Discussion

TFs or miRNAs usually co-regulate with each other to control the expression of several genes. This suggests that the topological properties of regulators could be related with those functions. In the present study, we compared four topological properties of different types of regulator and an interesting pattern in terms of how cells organize their regulatory information emerged.

The relative high TCR values of TS miRNAs suggest TS miRNAs tend to regulate different sets of targets, whereas trivial miRNAs are more likely to co-regulate the same set of targets. We also performed functional enrichment analysis of genes regulated by TS miRNAs and trivial miRNAs using the DAVID [27,28] online tools. For example, in brain, the targets of non-TS miRNAs were enriched in fundamental biological processes, such as metabolic processes (GO: 006259, GO: 0016071) and transport (GO: 0045031, 0051028), and their enriched KEGG (Kyoto encyclopedia of genes and genomes) pathways were “nucleotide excision repair” (hsa03420) and “citrate cycle” (hsa00020). As well as several fundamental biological processes, the targets of TS miRNAs were enriched in tissue specific functions, such as “neuropeptide receptor activity” (GO: 0008188), “motor neuron axon guidance” (GO: 0008045), “cell morphogenesis” (GO: 0000902) and the KEGG pathway “axon guidance” (hsa04360). These observations indicate that trivial miRNAs mainly regulate genes involved in fundamental cellular functions, while TS miRNAs tend to regulate genes with tissue-specific functions. This implies that non-TS miRNAs form the core of human transcriptional regulation networks, while tissue-specific miRNAs are attached to the core at more peripheral positions of the networks, and form new regulatory modules to execute particular functions in tissue differentiation.

TS TFs tend to exhibit higher in-degree, out-degree, betweenness and cluster coefficient values than trivial TFs in four of the tissues examined. These results mean that TS TFs occupy important locations in the regulatory networks and act as message transmitters in the information flow from upstream activators to downstream effectors. This may help in the understanding of how different tissues achieve specificity. First of all, TS TFs are essential for tissue differentiation; second, TS TFs alone are not enough for tissue differentiation and co-regulation with other regulators is required. This complicated and finely tuned regulatory mechanism can ensure correct cell morphogenesis and tissue differentiation.

HK TFs tend to have neighbours that densely interact with each other, indicating that HK TFs are inclined to form complicated associations with other TFs and miRNAs in the expression of genes.

Disease regulators also show particular properties in the regulatory networks. Although no tendency of disease TFs/disease miRNAs to be either TS TFs/TS miRNAs or HK TFs/trivial miRNAs was found, disease TFs tend to be connected with small clusters and disease miRNAs show higher out-degree values than non-disease miRNAs. Some approaches for identifying disease genes based on topological properties have been shown effective in protein-protein interaction network [11,29-31]. Applying the methods to transcriptional regulatory network may lead new disease genes and miRNAs to be found. These topological differences between disease and non-disease regulators may also provide a way to find drug targets for early diagnosis and treatment .

We also calculated characteristic path length, average in-degree, average out-degree, average betweenness and average cluster coefficient for seven tissues (see Table S8 in Additional file 3). The characteristic path length, average in-degree and average out-degree are similar in brain and testis. The two tissues were found to be clustered together according to a bower-tie structure [22] previously. On the other hand, brain and testis were reported to have similarities in both mRNA and miRNA expression profiles [32,33]. This consistency between the network status and expression profiles for brain and testis may suggest some correlations between them.

It should be noted that there are some limitations of this study. The target genes of TFs and miRNAs in our TRNs are all come from prediction which may lead to some false positive. In addition, we did not subdivide the tissue to certain cell type when we constructed tissue TRN, which may limit the precision of the networks. Despite such limitations, the work provides a new sight into the topological properties of HK/TS regulators and disease genes. The results uncovered here are important for understanding the key roles of different types of regulators in normal and disease tissues.

## Conclusions

The study provides an insight into the regulatory patterns of different types of genes in tissue regulatory networks. Tissue-specific genes and disease genes have particular topological properties in transcriptional regulatory networks and certain topological properties relate to certain regulatory mechanisms in gene regulation. From the regulatory differences between TS and trivial miRNAs, we inferred that trivial miRNAs might act at the core of human transcriptional regulation networks to perform basic biological functions, while tissue-specific miRNAs might be attached to the core at more peripheral positions, and form new regulatory modules to execute particular functions in tissue differentiation. The higher in-degree, out-degree, cluster coefficient and betweenness values for TS TFs in four tissues indicated that TS TFs act as bridges in gene expression information flow from upstream activators to downstream effectors, which in turn contributes to differentiation of certain tissues. Last, disease TFs/miRNAs show topological differences to non-disease TFs/miRNAs. These differences may provide a way to find new disease genes that are important in the early diagnosis and prevention of disease.

## Methods

### Construction of tissue regulatory network

The tissue regulatory networks (TRNs) for the seven human tissues, brain, heart, kidney, liver, ovary, spleen and testis, were generally constructed in the following way. First, a reference network was constructed by predicting the regulatory relationships between TFs, miRNAs and non-TF protein genes throughout the whole human genome. Then, the TRN for a certain tissue was built by extracting the TFs, miRNAs and non-TF protein genes that are known to be expressed in that tissue and incorporating the regulations between them from the reference network.

For the reference network, the regulations between TFs and their targets were predicted by identifying the 5-way (human, dog, cow, mouse and opossum) conserved potential TF-binding sites within a 1-kb region upstream of each target gene with the help of the TRANSFAC database [34]. The regulations between miRNAs and the targets were predicted with Targetscan [35], Pictar [36] and Tarbase [37]. The intersection of the predicted results was finally integrated into the reference network. More detailed information concerning TRN construction is described in our previous study [22].

The gene expression data for protein-encoding genes (including TFs and non-TFs) were collected from UniGene [38] and CGAP [39], and the miRNA expression data were obtained from Landgraf et al. (2007) [40].

### Housekeeping, tissue-specific and disease genes

In general, HK genes are widely expressed in various types of tissue, while tissue-specific genes are expressed and function in one or several tissues/cell types. If a TF is widely expressed, we name it HK TF. Similarly, if a TF is specifically expressed, we call it TS TF. For miRNA, we define it as TS miRNA if it is specifically expressed in one tissue. The other miRNAs are the type of trivial miRNAs.

- HK gene information was collected from Eisenberg et al. [41] and Chang CW et al. [42], while TS gene from the TiGER database [43] and Dezso Z et al. [19]. In total, 2827 HK genes and 4897 TS genes were recognized. 95 TS miRNAs were obtained from the literature [40]. In addition, 7877 disease genes and 317 miRNAs were acquired from the OMIM [44] and miR2Disease databases [45], respectively.

### Calculation of topological difference

For two sets of genes (e.g. TS TFs and trivial TFs, i.e. non-TSs and non-HK TFs) in a certain TRN, the topological difference between them is calculated as follows. First, we calculated the average of the topological parameter (i.e. in-degree, out-degree, cluster coefficient, or betweenness) for these small sets of genes. Second, 2000 random sets were generated to represent the large set of genes. Each random set is produced by randomly picking out the same number of genes as are in the small set from the large set of genes. Third, the average topological parameter of each random set was calculated. Finally, the average value of the small set was compared with those of the 2000 random sets, and the probability that the value of the small set is larger or less than that of the random set gives the P-value that characterizes the topological difference between these two sets of genes.

Note that a P-value < 0.10 indicates significant topological difference.

## Competing interests

The authors declare that they have no competing interests.

## Authors’ contributions

PL, JL and JW conceived the study; PL, ZZ, XH and JL carried out the analysis; PL wrote the manuscript. JL and JW supervised the work. All authors have read and approved the final manuscript.

## Supplementary Material

Additional file 1 Fisher exact test for tissue specific, house-keeping and disease TFs in seven tissues. Click here for file

Additional file 2 Number of disease TFs and miRNAs. Click here for file

Additional file 3 Network properties for seven tissues. Click here for file
