# Supplementary material for: Characterization of regulatory features of housekeeping and tissue-specific regulators within tissue regulatory networks
Source: BMC Syst Biol. 2013 Oct 31;7:112. doi: 10.1186/1752-0509-7-112 (PMC3843562; doi:10.1186/1752-0509-7-112)
Supplement: Additional file 1 — Fisher exact test for tissue specific, house-keeping and disease TFs in seven tissues. [file 1752-0509-7-112-S1.docx]

**Additional file 1**

**Table S1.****p value for fisher exact test**

|  | **brain** | **heart** | **kidney** | **liver** | **ovary** | **spleen** | **testis** |
| --- | --- | --- | --- | --- | --- | --- | --- |
| **P value** | 6.67E-31 | 0.086644 | 3.85E-33 | 1.18E-10 | 1.73E-25 | 6.38E-15 | 1.14E-09 |

**Table S2. p value for fisher exact test**

|  | **brain** | **heart** | **kidney** | **liver** | **ovary** | **spleen** | **testis** |
| --- | --- | --- | --- | --- | --- | --- | --- |
| **P value** | 1.79E-08 | 0.583651 | 5.90E-12 | 3.55E-06 | 5.05E-14 | 2.15E-12 | 3.43E-07 |

**Table S3. p value for fisher exact test**

|  | **brain** | **heart** | **kidney** | **liver** | **ovary** | **spleen** | **testis** |
| --- | --- | --- | --- | --- | --- | --- | --- |
| **P value** | 2.91E-69 | 3.86E-31 | 6.27E-47 | 5.11E-28 | 1.81E-48 | 1.97E-40 | 3.25E-50 |

**Table S4. p value for fisher exact test**

|  | **brain** | **heart** | **kidney** | **liver** | **ovary** | **spleen** | **testis** |
| --- | --- | --- | --- | --- | --- | --- | --- |
| **P value** | 2.37E-30 | 1.21E-12 | 4.18E-18 | 1.17E-17 | 9.70E-22 | 8.63E-25 | 1.93E-24 |

**Table S5. p value for fisher exact test**

|  | **brain** | **heart** | **kidney** | **liver** | **ovary** | **spleen** | **testis** |
| --- | --- | --- | --- | --- | --- | --- | --- |
| **P value** | 3.76E-65 | 4.19E-15 | 1.49E-49 | 2.00E-21 | 2.47E-23 | 2.25E-35 | 8.39E-33 |

**Table S6. p value for fisher exact test**

|  | **brain** | **heart** | **kidney** | **liver** | **ovary** | **spleen** | **testis** |
| --- | --- | --- | --- | --- | --- | --- | --- |
| **P value** | 2.17E-51 | 2.34E-22 | 5.10E-49 | 1.05E-25 | 2.01E-34 | 1.44E-28 | 1.60E-39 |
