# Supplementary material for: Characterization of regulatory features of housekeeping and tissue-specific regulators within tissue regulatory networks
Source: BMC Syst Biol. 2013 Oct 31;7:112. doi: 10.1186/1752-0509-7-112 (PMC3843562; doi:10.1186/1752-0509-7-112)
Supplement: Additional file 2 — Number of disease TFs and miRNAs. [file 1752-0509-7-112-S2.docx]

**Additional file 2**

**Table S7.** **Number of disease TFs and disease miRNAs**

|  | **Disease TF** | **Disease non-TF** | **Disease miRNA** | **Total miRNA** |
| --- | --- | --- | --- | --- |
| **Brain** | **119** | **1223** | **128** | **148** |
| **Heart** | **82** | **658** | **51** | **54** |
| **Kidney** | **68** | **558** | **114** | **130** |
| **Liver** | **59** | **539** | **65** | **68** |
| **Ovary** | **112** | **861** | **78** | **86** |
| **Spleen** | **103** | **834** | **75** | **80** |
| **Testis** | **170** | **1366** | **91** | **105** |
