# Supplementary material for: Characterization of regulatory features of housekeeping and tissue-specific regulators within tissue regulatory networks
Source: BMC Syst Biol. 2013 Oct 31;7:112. doi: 10.1186/1752-0509-7-112 (PMC3843562; doi:10.1186/1752-0509-7-112)
Supplement: Additional file 3 — Network properties for seven tissues. [file 1752-0509-7-112-S3.docx]

**Additional file 3**

**Table S8.** **Network properties for seven tissues**

|  | **average**  **in-degree** | **average**  **out-degree** | **average**  **cluster coefficient** | **average**  **betweenness** | **characteristic path length** |
| --- | --- | --- | --- | --- | --- |
| **brain** | 8.860635 | 8.860635 | 0.009473 | 1.08E-05 | 17.705 |
| **heart** | 4.98306 | 4.98306 | 0.010913 | 1.75E-05 | 9.957 |
| **kidney** | 7.451969 | 7.451969 | 0.007099 | 4.49E-05 | 14.891 |
| **liver** | 5.641883 | 5.641883 | 0.010876 | 3.52E-05 | 11.274 |
| **ovary** | 6.518187 | 6.518187 | 0.011158 | 1.71E-05 | 13.021 |
| **spleen** | 6.321903 | 6.321903 | 0.008791 | 1.90795E-05 | 12.627 |
| **testis** | 8.526434 | 8.526434 | 0.0107 | 8.41401E-06 | 15.306 |
